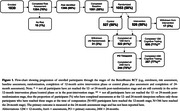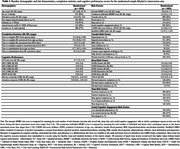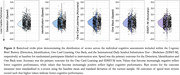# Baseline demographic, risk and cognitive characteristics of the final randomized sample enrolled in a decentralised multi‐domain lifestyle intervention trial (BetterBrains) to delay cognitive decline

**DOI:** 10.1002/alz.090564

**Published:** 2025-01-09

**Authors:** Emily Rosenich, Stephanie Pirotta, Hannah Cummins, Maya Norfolk, Gabrielle Da Costa, Nawaf Yassi, Leonid Churilov, Richard O. Sinnott, Amy Brodtmann, Matthew P. Pase, Chris Barton, Ashley I. Bush, Paul Maruff, Darshini Ayton, Yen Ying Lim, BetterBrains Research Group

**Affiliations:** ^1^ Turner Institute for Brain and Mental Health, Monash University, Melbourne, VIC Australia; ^2^ Monash University, School of Public Health and Preventative Medicine, Melbourne, VIC Australia; ^3^ The Walter and Eliza Hall Institute of Medical Research, Parkville, VIC Australia; ^4^ The Royal Melbourne Hospital, Parkville, VIC Australia; ^5^ The University of Melbourne, Melbourne, VIC Australia; ^6^ University of Melbourne, Melbourne Australia; ^7^ Cognitive Health Initiative, Central Clinical School, Monash University, Melbourne, VIC Australia; ^8^ Harvard T.H. Chan School of Public Health, Harvard University, Boston, MA USA; ^9^ Turner Institute for Brain and Mental Health & School of Psychological Sciences, Monash University, Clayton, VIC Australia; ^10^ Monash University, Melbourne, VIC Australia; ^11^ The Florey Institute of Neuroscience and Mental Health, The University of Melbourne, Australia, Melbourne, VIC Australia; ^12^ Florey Institute of Neuroscience and Mental Health, Parkville, VIC Australia

## Abstract

**Background:**

The BetterBrains Trial is a prospective behavior‐modification blinded endpoint randomized controlled trial to delay cognitive decline in middle‐aged adults (aged 40‐70) with a family history of dementia. The primary outcome is absence of decline on at‐least one out of four cognitive tests at 24‐months. We present trial recruitment and current participant completion statistics and baseline demographic, modifiable dementia risk factor (MDRF) and cognitive characteristics of the randomized sample, blinded to intervention arm.

**Method:**

Participants completed online assessments (betterbrains.org.au), including assessment of vascular, sleep, mood, and social/cognitive engagement MDRFs. Participants with ≥1 MDRF were eligible. Participants also completed assessments of cognition, general health, medical history, and lifestyle factors at baseline, 12‐ and 24‐months. Unsupervised cognitive testing was conducted using the Cogstate Brief Battery (see Figure 2 for details). Subjective cognition was assessed using a modified Cognitive Function Instrument.

**Result:**

From August 2021 to July 2023, 1830 participants enrolled and 1053 (57%) were randomized (Figure 1). Randomized participants were on average 59.7 (±6.8) years, 83% women, 90% highly educated (≥12 years of education), 93% White, and 86% reported a first‐degree family history of dementia (Table 1). Importantly, 27% resided in regional or rural Australia (Table 1). Participants reported an average of 6.5 MDRFs (max = 18) and a mean Cardiovascular Risk Factors, Aging and Incidence of Dementia risk score (without *APOE*) of 6.3 (max = 15) (Table 1). Baseline cognitive performance scores are presented in Table 1 and Figure 2. Randomized participants required, on average, 19.2 days to complete baseline assessment. To date, 61% and 70% of randomized participants have completed the 12‐ and 24‐month assessments (respectively), with study attrition at 3% (Figure 1).

**Conclusion:**

Compared to the general population, participants in this decentralised trial exhibit some increased risk of dementia, indicated by high prevalence of first‐degree dementia family history, female gender, and prevalent MDRFs. Participant characteristics are similar to those observed within other lifestyle intervention trials. The finding that 27% of the sample resides in a regional or rural area highlights that the BetterBrains methodology facilitates inclusion of hard‐to‐reach populations. Low attrition and high follow‐up rates suggest that the BetterBrains program is acceptable and feasible.